# Dos and Don’ts in Kidney Nutrition: Practical Considerations of a Panel of Experts on Protein Restriction and Plant-Based Diets for Patients Living with Chronic Kidney Disease

**DOI:** 10.3390/nu17122002

**Published:** 2025-06-14

**Authors:** Massimo Torreggiani, Carla Maria Avesani, Barbara Contzen, Adamasco Cupisti, Sylwia Czaja-Stolc, Claudia D’Alessandro, Liliana Garneata, Abril Gutiérrez, Françoise Lippi, Carmen Antonia Mocanu, Alice Sabatino, Giorgina Barbara Piccoli

**Affiliations:** 1 Néphrologie et Dialyse, Centre Hospitalier Le Mans, 72037 Le Mans, France; flippi@ch-lemans.fr (F.L.); gbpiccoli@yahoo.it (G.B.P.); 2 Division of Renal Medicine, Baxter Novum, Department of Clinical Science Intervention and Technology, Karolinska Institutet, 14186 Stockholm, Sweden; carla.avesani@ki.se (C.M.A.); alice.sabatino86@gmail.com (A.S.); 3 NephroLogik—Ernährungstherapie bei Chronischer Niereninsuffizienz, 51465 Bergisch Gladbach, Germany; info@nephrologik.de; 4 Division of Nephrology, Department of Clinical and Experimental Medicine, University of Pisa, 56126 Pisa, Italy; adamasco.cupisti@unipi.it (A.C.); claudia.dalessandro@unipi.it (C.D.); 5 Department of Clinical Nutrition and Dietetics, Faculty of Health Sciences, Medical University of Gdansk, 80-211 Gdansk, Poland; sylwiaczajastolc@gmail.com; 6 Department of Internal Medicine and Nephrology, Carol Davila University of Medicine and Pharmacy, 050474 Bucharest, Romania; lilianagarna@yahoo.com (L.G.); carmen_a9@yahoo.com (C.A.M.); 7 Sociedad Mexicana de Etudios en Ciencias de la Salud, Mexico City 06760, Mexico; abril.gutierrez@fresenius-kabi.com

**Keywords:** low-protein diets, protein intake, elderly patients, ketoanalogues, ultra-processed food, education

## Abstract

Dietary management is a pillar of chronic kidney disease (CKD) treatment. While some rules are the same as dietary prescriptions for the general population and those suffering from other chronic diseases (energy intake, salt intake, avoidance of ultra-processed food and limited intake of animal fats), in non-dialysis-dependent patients living with CKD, the specific focus is on protein intake. Low-protein diets (LPDs) and supplemented very low protein diets (sVLPDs) have been successfully employed to decrease the symptoms of people living with non-dialysis-dependent CKD, delay the progression of the disease and retard the need for dialysis. Randomized clinical trials have yielded conflicting results on efficacy, resulting in conflicting guidelines. Concerns about the risk of malnutrition (specifically when the main source of proteins is plant-derived), electrolyte imbalances, and energy intake, and the idea that adherence is difficult, jeopardize the use and wide application of LPDs and sVLPDs. That dietary management focuses mainly on nutrients while dietary quality occupies second place is also an erroneous concept that requires discussion. In September 2023, a group of experts composed of nephrologists and dieticians gathered in Frankfurt, Germany, to try to reconcile the different guideline indications and address most of the common doubts of final dispatchers to increase the prescription of “renal diets” and improve people living with CKD’s adherence to them.

## 1. Introduction

Dietary management is a pillar of the conservative management of chronic kidney disease (CKD). The observation that most of the body’s nitrogenous wastes are produced by the catabolism of dietary proteins, and that the burden of acid and phosphorus that need to be excreted comes from animal foods, led Thomas Addis to propose low-protein diets (LPDs) to treat people living with CKD as early as the 1930s [1]. Since then, a large body of literature has explored the effects of LPDs and very low-protein diets (VLPDs) supplemented with a mixture of essential amino acids (EAAs) and keto acids (KAs) on the progression, symptom control, and need for dialysis in people living with CKD.

In 2020, the US National Kidney Foundation Kidney Disease Outcomes Quality Initiative (NKF KDOQI) issued international guidelines on the dietary management of CKD that recommended that all people living with CKD, starting from stage 3, should receive a protein-restricted diet (LPD, about 0.6 g of proteins per kg of ideal body weight per day, or VLPD, 0.3–0.4 g/kg/day) to delay progression to kidney failure, mitigate the metabolic derangements that occur as kidney function decreases, and reduce the risk of death. The evidence on which the recommendations were based was rated 1A, i.e., the highest level of certainty of evidence [2].

This guideline goes beyond the classic statements that a low-protein diet (LPD) starts at 0.8 g of proteins/kg/day, of which at least 50% should be high-quality protein (animal-derived proteins), by acknowledging the recent reduction in recommended daily allowances, identifying in a diet with a protein content 0.8 g/kg/day a “normalized” diet in terms of protein intake, and stating that there is no evidence favoring animal versus plant derived proteins, provided essential amino acid intake is granted.

In the same guidelines, the attitude towards protein reduction in diabetic patients was more moderate, suggesting normalization or a decrease to 0.6 g/kg/day of proteins, provided that, for diabetic and non-diabetic patients, energy intake is sufficient [2].

Conversely, the most recent guidelines on the management of CKD, Kidney Disease: Improving Global Outcomes (KDIGO), issued in 2024, dampened enthusiasm for LPDs, suggesting that people living with CKD stages 3 to 5 that are not on dialysis should be managed with a diet with a protein intake corresponding to the recommended daily allowances (RDA) for the general population, i.e., 0.8 g/kg of body weight/day and that low-protein diets and VLPDs should be prescribed only to highly motivated people living with CKD at risk for progression to kidney failure [3].

Although both guidelines are based on the same randomized controlled trials (RCTs), KDIGO rated the evidence to be of relatively low quality, leading to a much weaker recommendation (2C, i.e., an expert suggestion because of the low certainty of the evidence presented). Different goals (focusing only on nutrition vs. comprehensive evaluation of CKD treatments) and specific expertise may have played a role in the different interpretation of the grade of evidence.

The confusion is increased by the fact that the KDIGO guidelines do not contain any indications on using moderately restricted LPDs and, while suggesting “normalization” to the RDA of 0.8 g/kg/day, also state that only people with potentially progressive CKD should avoid following a diet with a protein intake above 1.3 g/kg/day.

Although both guidelines agree that very high protein content should be avoided in the diet of people living with CKD, and that only metabolically stable people should be prescribed an LPD or a VLPD, the discrepancy between the recommendations testifies to the divergencies that make it difficult to reach a consensus. This contributes to uncertainty and to differences in clinical practice in the dietary management of people living with CKD. In addition, traditional prejudices against LPDs and VLPDs are slow to expire and further limit the application of these interventions, mainly for fear of inducing protein-energy wasting (PEW) and of negatively affecting people’s quality of life (QoL) [4].

In spite of these concerns, an increased risk of PEW was not reported in large series and RCTs on diet (possibly also as a result of the selection of the settings of study, and of the exclusion of malnourished patients from dietary restrictions). As for QoL, the KDOQI guidelines recognize the lack of extensive studies on this issue. Two large multicenter studies, in a context of a patient-centered, flexible dietary approach, failed to associate dietary prescription with QoL [5,6,7].

To find ways to mediate between these different indications, in September 2023, a group of experts met in Frankfurt to discuss the practical implementation of a protein-restricted diet for people living with CKD, while maintaining adequate dietary quality, and allowing patients to participate in the decision-making process in order to provide some insights to nephrologists and dieticians without specific experience on these issues. This narrative review is a summary of that discussion.

## 2. General Issues

One of the more controversial points of the different recommendations is the level of evidence. Both the KDOQI and the KDIGO guidelines select RCTs only and adopt the GRADE method for determining the quality of evidence but, as discussed above, the authors recognize very different strengths and certainty levels [8]. This is probably due to the fact that few RCTs have assessed the efficacy of LPDs or VLPDs in delaying the progression of CKD or the need for dialysis and most of them suffered either from an adherence bias (the Modification of Diet in Renal Disease study, for example, had conflicting results when its analysis was performed per protocol or per intention to treat) or from a selection bias (in the study by Garneata and coworkers, about 200 people living with CKD were enrolled out of the 1400 assessed for eligibility), that prevent the generalizability of their results [9,10,11,12].

Along these lines, the KDIGO guidelines acknowledge that their recommendations sought to avoid the challenges associated with adherence to LPD and the risk of PEW [3].

Both guidelines agree that people empowerment is vital to the success of an LPD and that the dietary management of CKD should be multidisciplinary, including at least one nephrologist and one trained dietician, an option that is not universally available [2,3,13]. However, in a patient-centered context, these concerns should not be an excuse for not providing people with CKD with dietary management, including the option of protein-restricted diets.

Unlike RCTs, several observational studies have described the benefits of a reduced protein intake in people living with CKD [14]. While observational studies cannot firmly establish efficacy, they do allow us to study outcomes that are not assessable in randomized trials for ethical reasons (such as nutritional management in pregnancy) and enable us to implement flexible stepwise approaches to dietary protein restriction that could improve patients’ adherence [15].

Indeed, the concerns regarding malnutrition are not supported by RCTs or large observational studies, provided that patients are monitored and that, whenever the protein intake is decreased below 0.6 g/kg/day, the diet is supplemented with essential amino acids and ketoacids (see specific paragraph below).

**Figure 1:**
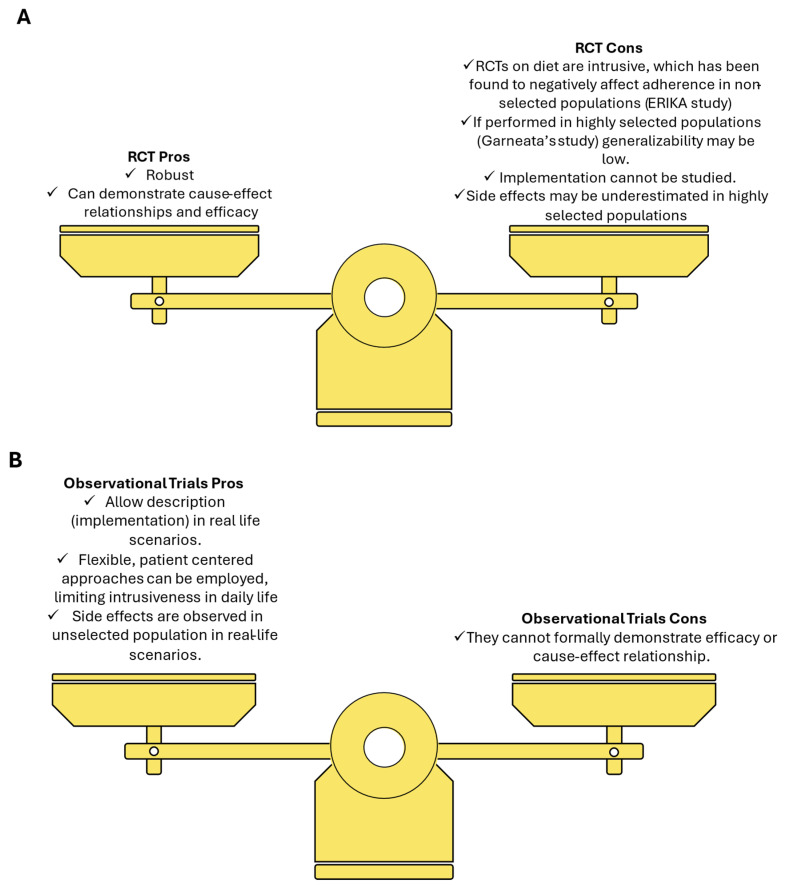
Balancing study designs in nutritional management of CKD: (**A**) randomized clinical trials (RCT); (**B**) observational studies.

Given how difficult it has been to perform RCTs on dietary management in CKD, the most recent systematic Cochrane review on LPDs for people living with CKD with non-diabetic CKD, published in 2020, included fewer than 3000 participants, suggesting that other types of studies are needed to gain insight into the delicate matter of dietary management of the 10% of the population which is affected by CKD [16] (Figure 1).

Target trial emulation or patient preference trials may be valid alternatives, as they, by respecting patients’ choices, are compatible with unselected, real-life assessment. However, they are more complex and may be more expensive to carry out [17,18].

## 3. The Animal vs. Vegetal Protein Conundrum

In the past, it was held that in low-protein diets for people living with CKD at least 50% of the proteins should be of animal origin [19,20,21,22,23,24]. Animal-derived proteins contain all the essential amino acids that humans cannot synthetize in adequate amounts (hence the term high biological value), and more efficiently preserve the body’s protein pool; they contain a greater amount of sulphurated amino acids [24]. However, the growing interest in plant-based diets has recently changed this paradigm, as a diet mainly or entirely composed of plant-origin food is now recognized as a feasible option in health and in several chronic diseases, including CKD [25]. A recent study showed a reduction of 4% in the risk of developing CKD for every 0.1 g/kg/day intake of plant-derived proteins in the general population [26]. Specifically, in nephrology, a plant-based diet is the only strategy that makes it possible to prescribe a very low protein intake, i.e., 0.4 g/kg of body weight/day or less [2]. Plant-based diets contain a higher amount of fiber and alkali than animal-based ones and are better at correcting the metabolic acidosis associated with CKD, and preventing intestinal dysbiosis [27]. It is important to clarify that plant-based diets are not vegan diets but instead comprise a group of dietary patterns in which most of the protein intake comes from plant-based sources, e.g., the Mediterranean diet, DASH (Dietary Approaches to Stop Hypertension), the Okinawan diet, PLADO (Plant Dominant Low-Protein Diet), and NNRD (New Nordic Renal Diet) (Table 1). Considering the metabolic benefits of such plant-based dietary patterns, the two recent guidelines on nutrition in CKD supported the prescription of plant-based diets to people living with CKD. Although they state that the current evidence is insufficient for recommending a specific source of protein, the KDOQI guidelines do not exclude this option, while those of KDIGO specifically recommend plant-based diets [2,3]. Nevertheless, some concerns remain about the use of plant-based diets, such as their high content of potassium (see below). The experts involved in this discussion agreed that there is room for implementing plant-based diets in people living with CKD, provided that people are monitored to avoid PEW and essential amino acid deficits. The latter may also be corrected using EAA and KA supplements, that are mandatory in people prescribed a VLPD.

Since plant-based diets may have a low content of some vitamins (B12 and D), and micronutrients (zinc, iodine, calcium), people should be periodically monitored [28]. For instance, a series of studies and a recent systematic review found that vitamin B12 intake in vegan people was far below the recommended intake (according to the systematic review: 0.24 to 0.49 mg/day vs. 2.4 mg/day) [29]. The same review reported discrepancies between real and recommended intake of vitamin D, zinc, iodine, and selenium among vegans [29]. Although these reduced intakes are not likely to cause major health issues, these observations set the ground for the panelists to agree that micronutrient blood levels should be monitored in people on a plant-based diets to promptly supplement and correct deficiencies.

In this review, we will further use the term animal-based diets to define omnivorous diets rich in animal-derived food.

Dos: Prescribe a plant-based diet in all CKD stages; regularly check nutritional status; periodically check for intake of all the essential amino acids in the same meal (ideally) or at least in the same day, in particular for those on vegan diets. Consider occasionally allowing unrestricted meals. Consider supplementation with EAAs and KAs for people on vegan diets, as this simplifies management (0.6 g/kg/day diets) and makes it possible to prescribe VLPDs.

Don’ts: Do not limit prescription for fear of PEW. Do not prescribe a plant-based (vegan) or a VLPD without a detailed monitoring plan.

**Table 1:** Characteristics and expected benefits of different diets in people living with chronic kidney disease (CKD).

Diet	Composition	Expected Benefits in CKD	
Mediterranean diet [30]	Predominance of vegetal and fish protein versus meat protein.		Reduced cardiovascular risk Lower mortality risk Reduced phosphate load Reduced infalmmation and oxydative stress Better microbiota regulation Lower CKD progression
Plant-dominant Low-protein diet (PLADO) [31]	0.6–0.8 g of proteins/kg/day, >50% plant-based sources.	Reduced acid load	
Okinawan diet [32]	Low caloric intake, predominance of sweet potatoes, green-leafy or yellow-root vegetables, and soy.		
Dietary Approaches to Stop Hypertension diet (DASH) [33,34]	High in fruits, vegetables, and low-fat dairy products, with reduced saturated and total fat.	Lower blood pressure	
Flexitarian [35]	Predominantly vegetarian, with occasional inclusion of animal products.		
New Nordic renal diet [36]	Predominant plant-based food, 0.8 g of proteins/kg/day, <5 g/day of sodium chloride.	Reduced proteinuria Reduced body weight Lower blood pressure	
Ketogenic diet [37,38]	Predominance of fat (>70%), proteins 6–20%, carbohydrates < 10%.	Improved glycemic control Weight loss Lower blood pressure Reduced inflammation Slower CKD progression Possible benefit in polycistic kidney disease	
Western diet [39]	Highly caloric, processed and refined foods, high content of sugars, salt, and fat and protein from red meat.	None: Increased proteinuria Faster CKD progression Increase inflammation and oxydative stress Intestinal dysbiosis Increased acid load Increased potassium and phosphorus load Increased salt load Increased blood pressure	

## 4. Should We Worry About Potassium?

Animal protein intake is of special concern because of the higher production of fixed acids, potentially enhancing CKD-related metabolic bone disease (CKD-MBD), and for its high phosphorus load. In addition, protein-derived peptides may modulate gut microbiota, while a high quantity of animal-based proteins induces hyperfiltration and may increase proteinuria. Conversely, one of the most frequently reported concerns with plant-based diets is their supply of a higher amount of potassium. Hyperkalemia is associated with weakness, paralysis, the development of cardiac arrythmias, and ultimately increased mortality [40,41]. However, the risk of hyperkalemia is blunted in plant-based diets by better correction of metabolic acidosis, faster intestinal transit time and lower bio-availability of potassium in plant-based, fiber-rich foods, that do not rely on ultra-processed food (UPF) [42]. In fact, alkalosis induces shifting K in the intracellular fluid and controlling the K serum levels [43,44]. Fiber promotes intestinal motility, reducing the absorption of K in the gut and favoring its fecal excretion [45]. Animal-based foods may be important contributors to dietary potassium intake due to their high potassium content, especially if enhanced with additives [46]. In addition, while K present in animal-derived foods has a 70–90% gut absorption rate bioavailability is only 50–60% in fruits and vegetables [47,48,49]. Moreover, carbohydrates contained in plant-derived food, through the stimulation of insulin secretion, may contribute to blunting the effects of dietary K on serum levels [50].

This is why the consumption of animal-derived foods does not eliminate the risk of hyperkalemia in people living with CKD [50]. Potassium bioavailability is, as previously discussed, higher in UPF due to food additives containing potassium [51]. It is therefore recommended that labels on all food products be checked to ensure that potassium intake from food additives is limited. Detailed information on the potassium content of different foods with visual handouts for patient’s education is available on the Dietary Guidelines for Americans website (https://www.dietaryguidelines.gov/food-sources-potassium, Accessed on 6 June 2025) or on the European Renal Association website (https://www.era-online.org/cookbook/materials/, Accessed on 6 June 2025). The panel would like to underline that there are no prohibited foods, but the dietary plan should be adapted to the patient’s baseline clinical features (for instance, the use of loop diuretics or potassium-sparing agents, angiotensin-converting enzyme inhibitors (ACEi), etc.), preferences and needs.

Wise choices of low potassium fruits and berries (like pineapples and blueberries) and, when needed, boiling or blanching, may allow further dietary freedom in selected cases prone to hyperkalemia.

In addition, there are other causes of hyperkalemia that are not dietary-related, namely metabolic acidosis, intestinal constipation, and use of (ACEi). Given this background, it is not surprising that dietary K intake does not correlate with serum K levels, and this shows that the claim that an adequate intake of fruit and vegetables is the main cause of hyperkalemia in people living with CKD is unfounded [52]. Potassium intake widely varies worldwide, being lower in North America and Asia and higher in European countries [53,54]. Potassium intake is, however, usually calculated using food diaries that do not consider added potassium in processed foods and UPFs. Although recent evidence challenged the role of dietary potassium in the development of hyperkalemia, it is still suggested that individuals with an estimated glomerular filtration rate lower than 30 mL/min/1.73 m^2^ limit the daily intake to 3 g [55]. For this reason, as already mentioned, other causes of hyperkalemia should be considered before restricting potassium dietary intake. Nevertheless, the proportion of non-dialysis people living with CKD presenting hyperkalemia despite optimal nutritional treatment is not negligible and can be as high as 33% [56]. New potassium-binding drugs may help control hyperkalemia without the limitations imposed by the older ones, namely sodium polystyrene sulfonate, to avoid ominous side effects such as intestinal necrosis [57,58]. A recent study demonstrated that sodium zirconium cyclosilicate was effective in controlling serum potassium levels in people living with CKD consuming a plant-based diet with no need to change the dose of ACEi, allowing for a healthier diet prescription [59]. Furthermore, the experts agreed to highlight a note of caution regarding the new drugs available to delay the progression of CKD, such as mineral corticoid receptor agonists, as their use is not devoid of the risk of hyperkalemia [60]. Instead, sodium-glucose transporter 2 inhibitors do not cause hyperkalemia, and may have a synergistic effect when used with LPDs [61].

Dos: Prescribe plant-based diets in all CKD stages with regular checks of potassium and bicarbonate levels. Add potassium binders if they are needed, provided that food sources are controlled (readily absorbed potassium salts are ubiquitous additives in UPF; they are not always listed on food labels).

Don’ts: Do not limit prescription of plant-based diets in the case of high potassium levels, or fear of causing them. Do not prescribe a plant-based (vegan) or a VLPD without preparing a strict monitoring plan.

## 5. Phosphorus and the Diet

Calcium-phosphorus metabolism is altered in CKD, especially in its advanced stages, and, usually, compensatory mechanisms fail to maintain homeostasis as the glomerular filtration rate falls below 30 mL/min [62]. CKD-MBD is associated with vascular calcifications, stiffness and besides the alteration of bone metabolism, high phosphate and parathyroid hormone (PTH) levels are closely associated with cardiovascular and all-cause mortality [63]. Moreover, excessive bone reabsorption in CKD may significantly contribute to high phosphate serum levels [64]. Since the correlation between dietary intake and serum phosphate levels is well acknowledged, the classic therapeutic strategy to control phosphate levels is to counsel a diet providing phosphorus (P) intake below 800–1000 mg/day [65]. However, different sources have different bioavailability: organic P has a bioavailability of about 40–60% in animal-derived foods and only 20–40% in plant-derived ones, in which it is mainly in phytate form [66,67]. Conversely, the absorption of inorganic P contained in food preservatives and additives is from 90 to 100% [67]. In this context, even when relatively rich in P, plant-based diets, given their lower P bioavailability [68], are useful, while, as previously stated, UPF should be limited.

Some authors have suggested that P intake should be modulated according to kidney function [69]. However, alterations in P metabolism may begin long before an increase in serum phosphate levels becomes evident [70]. Thus, the experts involved in this discussion agreed that careful monitoring of P balance is needed even before the onset of hyperphosphatemia. Indeed, serum and urinary P levels should guide dietary management, adapting intake to the individual person. The American Kidney Fund have compiled a list of the phosphorus content of different foods which can be useful for healthcare providers as well as patients as educational or empowering material (https://kitchen.kidneyfund.org/wp-content/uploads/2024/08/Phosphorus-Food-Guide_FINAL_072424.pdf, Accessed on 6 June 2025). Useful educational leaflets can also be found on the European Renal Association website (https://www.era-online.org/cookbook/materials/, Accessed on 6 June 2025).

Dos: Prescribe plant-based diets as they are a source of P whose bioavailability is lower than in animal-based diets. Beware of UPF, since readily absorbed phosphate-based additives are ubiquitous in UPF and they may not be listed on the product labels.

Don’ts: do not limit prescription of plant-based diets in the case of high phosphate levels, or fear of causing them.

## 6. An Underestimated Problem: Establishing the “Ideal” Body Weight

**Figure 2:**
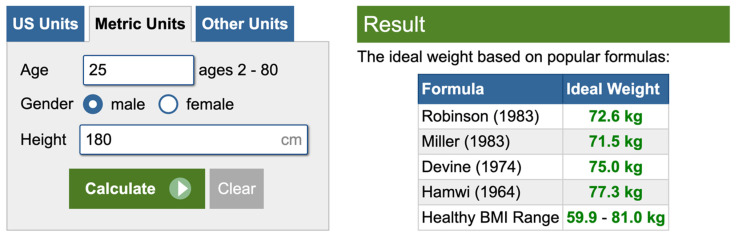
Calculating ideal body weight according to different formulas. Ideal weight calculator, available at https://www.calculator.net/ideal-weight-calculator.html.

Obesity represents a risk factor for CKD and its prevalence among people living with CKD can reach over 50% and up to 40% of people living with kidney failure [71]. Thus, when prescribing an LPD to an obese person, the body weight on which protein intake should be calculated is of utmost importance. According to the current guidelines, the prescribed amount of proteins per day should be based on ideal body weight (IBW), usually defined as the weight associated with the lowest mortality for a given height, age, and sex [2]. However, in obese people, using the ideal body weight may result in a drastic reduction in protein intake compared to baseline that could, in turn, induce protein wasting and reduce the patient’s adherence to the dietary regimen because of the frustration people experience when not meeting a prescription. There are several formulas for calculating ideal body weight [72,73,74,75,76,77,78,79]; notably, they may yield very different results (Figure 2 and Table 2). The use of adjusted body weight (AjBW), i.e., an intermediate weight between the ideal and the actual one does not solve the problem, as, once more, several formulas exist [80]. The approach supported by the expert panelists is to use real body weight for people with a BMI < 30–35 kg/m^2^ and the AjBW for people with a higher BMI.

While the complex management of obese people living with CKD is beyond the scope of this review, in keeping with the guidelines’ indications, the experts agreed that management should be tailored to each person, despite the higher need for resources and time that this entails. In people on weight-loss diets, the prescription should be adapted to each newly achieved goal [15]. This would lead to increasing motivation and therefore greater adherence [15]. The authors recommended that a person’s weight history and perspectives (transplantation, for instance) should be taken into account when prescribing a dietary plan so that benefits and risks are balanced and the best possible results are attained.

Dos: Consider each person’s body composition, weight trajectory and perspectives when approaching the issue of real or ideal body weight. Consider real body weight at least below a BMI of 30 kg/m^2^. Evaluate obese people individually and review prescriptions in case of modifications of body weight.

Don’ts: do not use a formula to calculate ideal or adjusted body weight without a previous critical appraisal of the individual person.

## 7. Energy Intake and the Risk of Protein Energy Wasting, the Boogeyman of Dietary Management of CKD

**Figure 3:**
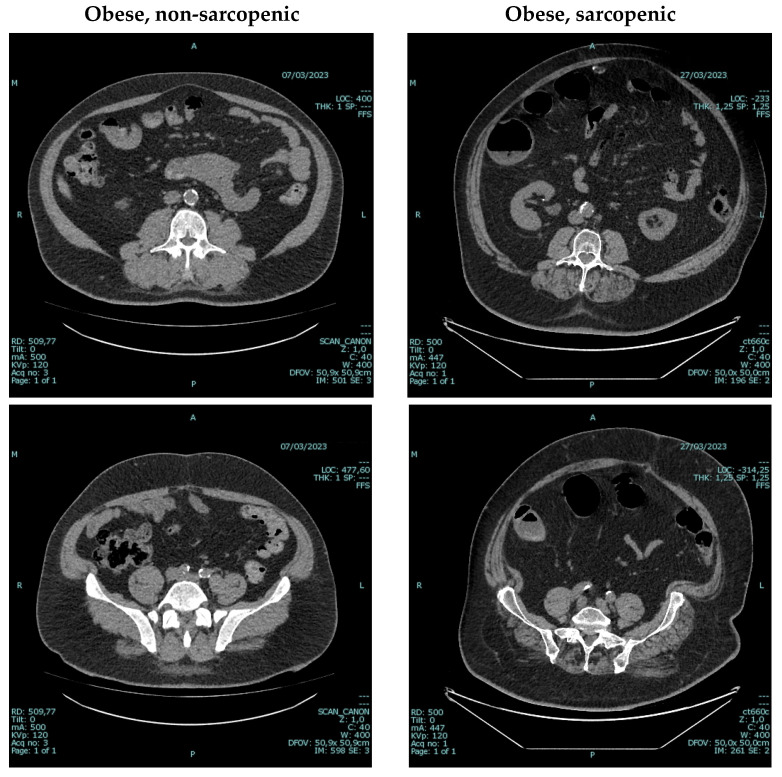
CT scan for the diagnosis of sarcopenic obesity. Legend: in addition to being used as traditional diagnostic tests, CT scans performed for other reasons provide valuable insights into the muscle mass of people living with CKD [81,82,83]. Free software exists to help interpret cross-sectional CT images, usually at the third lumbar vertebra (L3), or at the iliac crest, which can provide reliable estimates of body composition [84]. Notably, in the panel on the right, the quality and not only the quantity of muscle is impaired.

PEW is a clinical condition classically defined by the presence of three of the following criteria: loss of weight fat or muscle mass, low serum albumin, prealbumin or cholesterol, unintentional reduction in energy and/or protein intake [81]. The prevalence of PEW varies widely, but it is usually held that it affects up to 50% of people living with advanced CKD or kidney failure [82]. Interestingly, the phenotype of PEW is changing: the so-called skeleton man—young, thin, and wasted—in which PEW was caused by uremia, dietary over-restrictions, insufficient dialysis or a combination of the above, and in which increasing intakes allowed at least partial reversibility, has now been mainly replaced by a phenotype of sarcopenic obesity in obese, elderly people, usually with atherosclerosis and chronic inflammation, in which increasing intake is usually ineffective in restoring a positive anabolic balance [83,84]. For this reason, the importance of lab tests like albumin and prealbumin, which are useful but do not fully reflect the nutritional status, has been argued in favor of the criteria of unintentional loss of weight, and on assessment of muscle mass. Beyond traditional diagnostic tests, CT scans performed for other reasons, could provide valuable insights into the muscle mass of people living with CKD and help identifying sarcopenic–obese individuals (Figure 3) [84,85,86]. In this regard, plant-based diets, with their alkalinizing effect, may allow a better preservation of muscle mass [87,88,89].

The new KDOQI guidelines on nutrition in people living with CKD recommend an energy intake of 25–35 kcal/kg of ideal body weight/day, thus reducing the target from 30 to 35 kcal/kg/day, on account of the aging of the population [2,3]. This recommendation, however, does not take into consideration specific subpopulations like elderly inactive people or an extremely elevated BMI which may benefit from different energy intakes. Nonetheless, the range proposed by the KDOQI guidelines stresses the need for individualized approaches.

Maintaining an adequate energy intake considering the person’s lifestyle and daily activities is a priority not only so that PEW is avoided, but also because it makes it possible to avoid proteins being catabolized as energy sources in people on LPDs and VLPDs, with the consequent risk of negative nitrogen balance. “Energy first” should always be the starting point in treating people living with CKD.

LPDs and sVLPDs should provide enough energy to avoid PEW, but reconciling energy needs may not be easy, in view of the changing epidemiology of CKD, in the context of an aging world population and increased prevalence of obesity. CKD is predicted to be become the fifth cause of death by 2040, and future people living with CKD will be older, more fragile, and more difficult to manage [90,91,92].

Focusing on elderly people in order to decrease malnutrition, mortality, and frailty, geriatric societies and ESPEN recommend a protein intake of at least 1–1.2 g/kg/day, together with an energy intake corresponding to the renal guidelines (25–35 kcal/kg/day) [93]. In CKD, meeting these indications requires increasing energy from non-protein sources, a sometimes challenging task. Notably, recent studies refute the claim that elderly people have a spontaneously reduced protein intake, reporting in elderly people living with CKD a high prevalence of protein-rich diets, with an intake above 0.8 g/kg/day [94,95]. The proportion of elderly people starting dialysis is increasing: the median age at dialysis start is over 70 years in several European countries, with a global median of 68 years, and in several settings up to 15% of patients on dialysis are aged 80 years or more, while the proportion of patients aged over 90 is likewise increasing [96,97,98]. Elderly people (aged over 75 years) represent the major share of incident dialysis patients in the United States of America as well as in some European countries like France [99,100].

**Figure 4:**
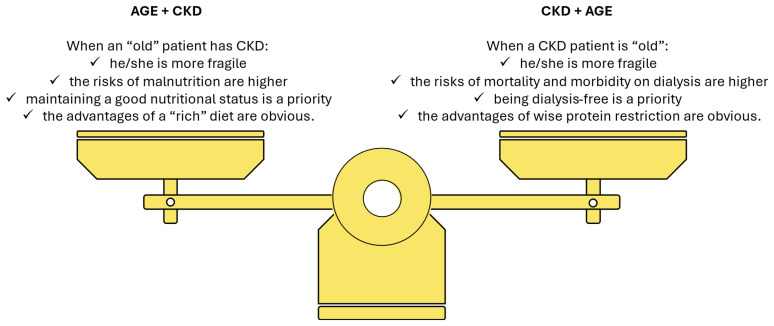
Balancing age and chronic kidney disease (CKD) (adapted from [101]).

Thus, the balance between prescribing LPDs, to retard, and ideally avoid dialysis start should be balanced against the fact that with an unrestricted diet could be easier to preserve or improve nutritional status in elderly people living with CKD. In this context, setting priorities is essential (Figure 4) [101].

According to the expert panel, while LPDs in well-nourished elderly people have the same indications as they do for the younger population, they should be considered with great caution and strict follow-up in people with low muscle mass, or impending signs of PEW, and, as previously stated, avoided in people PEW.

**Figure 5:**
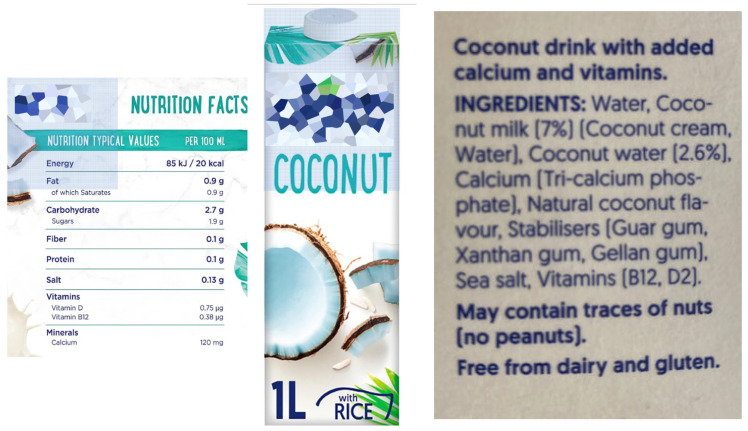
An example of a plant-based drink with high-energy and low-protein content.

**Figure 6:**
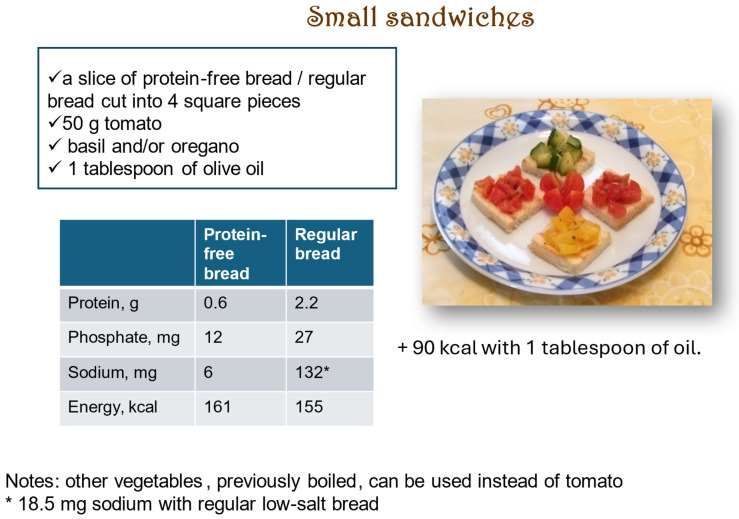
A recipe for a high-energy density snack with low-protein content.

Conversely, in individuals, mainly elderly, with CKD and low energy intake, when dietary management, including healthy energy dense food is not successful, oral supplements may be employed, at least temporarily. While most of the commercial oral nutritional supplements are both high in energy and in proteins, formulas containing high energy and low proteins are also available. Examples are plant-based drinks with a high caloric and low protein content (Figure 5) that can be used in creative ways (to make ice cream, for instance). Some of these drinks can also be easily home-made, although the energy and protein content of home-made drinks may be more difficult to estimate than those commercially available. As an example, according to some estimations, 100 mL of home-made almond milk would provide 3 times the amount of energy and 5.5 times the protein content than the same commercially available product [102]. Small energy-dense snacks eaten throughout the day are another option, eventually coupled with protein-free food in individuals with advanced CKD (Figure 6), providing energy without generating nitrogen waste. Liberal use of olive oil, and other vegetable oils (except for coconut and palm oil that need to be limited), may help increase energy intake (Figure 6). However, consider that vegetable servings, on which oil is frequently used, may increase the sense of satiety, and thus reduce energy intake.

Dos: When starting nutritional management of people living with CKD, always start from energy intake, modulating it according to nutritional status. A plant-based diet is compatible with high energy intake and can, when needed, be combined with energy-rich, low-protein supplements.

Don’ts: In people with evidence of PEW, do not restrict protein intake, mainly from vegetable sources, at least until energy needs are met. Avoid a high-protein intake to prevent protein hypercatabolism, potentially leading to acidosis, hyperkalemia, hyperphosphatemia and intestinal dysbiosis.

## 8. Very Low Protein Diets Plus Amino Acid and Ketoacid Supplements

While the two main current guidelines differ with respect to the role (if any) for the moderately restricted LPDs, they both agree on the fact that supplemented VLPDs (sVLPDs) have been shown to delay the need for kidney replacement therapy in selected, motivated and compliant individuals with advanced CKD [2,3,11,103]. Metanalyses show that sVLPDs are superior to LPDs in reducing progression to kidney failure [104]. VLPDs are, in essence, plant-based diets, and, as previously reported, need to be supplemented with EAAs and KAs to avoid protein wasting [11].

Shifting from an unrestricted diet to a sVLPD often requires a great leap and dietary restrictions may not meet patients’ actual preferences, even when motivation is high. This is why the expert panel suggested adopting a stepwise reduction in protein intake and emphasized that there are no predefined numbers to follow, and protein intake reduction should be a continuum from the baseline ideally to reach a level that allows stabilization of the kidney function, that could be set at different levels (normalization of protein intake, i.e., around 0.8 g/kg/day, moderate restriction, i.e., 0.6 g/kg/day) to reach in selected case a very low level of 0.3–0.4 g/kg/day, in the most motivated and adherent people living with CKD.

Severe protein restrictions should be considered in people with good nutritional status and good appetite who are willing to change their dietary habits. In people with malnutrition or/and severe inflammation, the causes should be considered before proposing any type of restriction.

Since one of the few large RCTs specifically focused on elderly people, and results were favorable, with no evidence of malnutrition, the panel felt that there is no age limit for using this approach [103].

Furthermore, considering that each reduction of at least 0.2 g/kg/day has a protective effect on kidney function and can retard the need for dialysis, a progressive reduction in protein intake is expected to provide at least some benefits even when the very low target is not reached [105]. While the interest of moderately restricted LPDs was recently challenged, the panel considers that they may allow stabilization of kidney function and are more easily managed at all ages. An added value is to prescribe a moderate protein restriction in the form of a plant-based diet, whose protein content is usually at or near the target of 0.8 g/kg/day. Plant-derived foods have substantial advantages compared to animal-derived ones, as previously discussed, and the change in dietary pattern may be advantageous by itself [106]. However, plant-based in general, and vegan diets in particular, can expose a person to the risk of selective essential amino acid deficiency. Hence, supplementation with EAAs and KAs is mandatory in VLPDS, and may in some selected cases be added to LPDs [107]. Through a transamination reaction KA can be converted into the corresponding essential amino acids without adding to the nitrogen pool in people living with CKD, according to some authors, acting as metabolic absorbents to reduce urea levels [107]. The only commercially available mixtures of EAAs and KAs in Europe contain the KA precursors of isoleucine, leucine, phenylalanine, valine, and methionine and the essential amino acids tryptophan, threonine, histidine, and tyrosine [107]. The total amount of EAAs and KAs per tablet is 630 mg. Since KAs are sold in the form of calcium salts (calcium content equal to 50 mg per tablet), they can induce hypercalcemia and gastrointestinal discomfort but also act as phosphate binders [107]. Furthermore, they contribute to the high pill burden of most people living with CKD and the number of pills and tablets a person is supposed to take is inversely associated with compliance [108].

While in VLPDs the agreed dose is one tablet for each 5 kg of body weight, there is no clear indication of the doses eventually needed to complement vegan or plant-based LPDs, in which they may be used to avoid selective essential amino acid deficiencies. The tablets should be taken and assimilated with the meals, and not with regular medications. It was the opinion of the expert panel that a lower KA posology can be used, in selected cases, in LPDs, to find a compromise between the benefits of the diet and the risk of PEW, as reported in clinical experiences [6,15]. The most frequently used dose both in the literature and according to the experts is one tablet per 8–10 kg of body weight [6,15,109].

Dos: Prescribe VLPDs to selected, motivated, and compliant people, both young and elderly living with CKD. These diets are in essence plant-based. Especially in people on unrestricted baseline diets, proceed stepwise, both with quality changes (from omnivorous to vegan) and protein intake reduction.

Don’ts: Do not prescribe a severe protein restriction without supplementation and without close monitoring. Do not prescribe sVLPD in malnourished people, especially if the etiology of PEW is uncertain. Do not use the same doses when supplementation is an “add-on treatment” in moderately protein-restricted diets. Consider a lower pill burden, instead.

## 9. Avoiding Problems: The Ultra-Processed Food Issue

In the modern era, time has become a luxury and cooking using fresh ingredients has become less common [110]. Fast foods and ready-to-eat dishes are appealing and tasty, and their use has exponentially increased in recent decades, especially in food deserts, i.e., areas characterized by low income and limited access to healthy food [111,112,113,114,115,116]. According to the NOVA classification ultra-processed foods (UPF) are “formulations of ingredients, mostly of exclusive industrial use, typically created by series of industrial techniques and processes” [117]. UPFs have been associated with an increased risk of cancer and cardiometabolic diseases as well as the rising prevalence of obesity [118,119]. Recently, there have been reports that also associated UPF consumption with the risk of developing CKD [120,121,122,123,124,125]. Several issues are connected to UPFs: they are energy-dense products, usually made using saturated fats and refined sugars; they contain a vast array of additives and preservatives containing potassium or phosphorus or sodium or a combination of more than one of these to enhance their palatability and conservation, while the amounts of fiber, vitamins, and antioxidants they contain is usually low due to changes in the food matrix that occur during industrial processing [126]. Moreover, while several additives have been associated with health risks, manufacturers are not obliged to declare the exact amount of additives a food contains [127]. Likewise, their content of added potassium or phosphorus is difficult to estimate. Finally, ready-to-eat foods do not benefit from alternative ways of preparing them, for instance to reduce potassium content: boiling in large quantities of water, and/or changing the cooking water two or three times, traditionally suggested to reduce the potassium and phosphorus content have little effect [128]. In the panel’s opinion, people living with CKD should be educated to recognize UPF and choose alternatives (see below). Few studies have proven the efficacy of shifting from a UPF-rich diet to freshly cooked meals. The example of a pregnant Mexican woman with CKD stage 5, who consumed a diet rich in ultra-processed foods, and who shifted to a plant-based LPD is one: despite late nephrology referral, she was able to give birth at term, without needing to dialyze, with a remarkable reduction in blood urea nitrogen levels [129]. Providing a healthy food basket to people living with CKD was effective in reducing UPF consumption and preliminary data suggest that stabilization of the kidney function can likewise be obtained [130].

Dos: encourage people living with CKD to pay attention to the quality of the food they eat and minimize the use of UPF.

Don’ts: do not reduce plant-based food, for example, in the case of hyperkalemia, until you have excluded regular use of UPFs and educated the people living with CKD to limit their use.

## 10. Education and Counseling as a Key to Success

**Figure 7:**
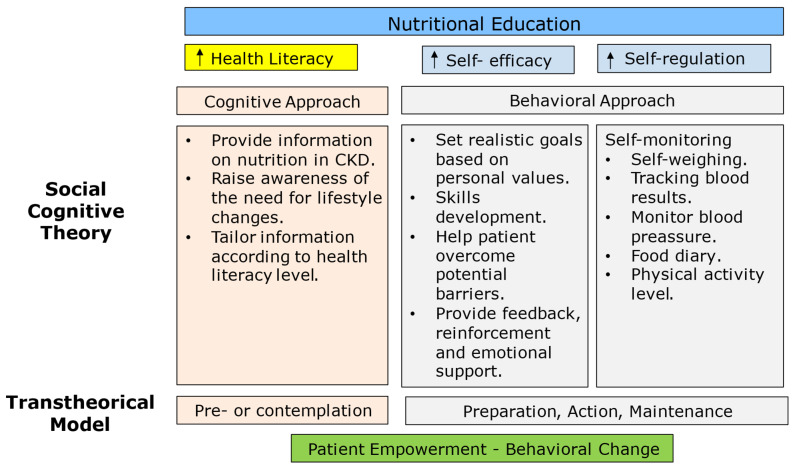
Cognitive and behavioral approaches in dietary education (adapted from [136]).

Education requires healthcare professionals’ time [131]. It has been estimated that, even in high intensity settings, physicians spend only 14.7% of their time in direct contact with the patients and more than 40% in their offices, for electronic medical record review and documentation [132]. With the overwhelming amount of paperwork doctors and nurses are confronted with, most complain about not having enough time to dedicate to education during regular consultations. Nevertheless, investing in nutritional education is pivotal for improving people empowerment and ameliorating compliance and finally outcomes [133,134,135]. In fact, adherence, compliance, and concordance, i.e., the modality of the patient–physician relationship, influence the results of the dietary management of CKD [13]. Cognitive and behavioral approaches eventually lead to people empowerment and behavioral changes (Figure 7) [136].

In the opinion of the expert panel, education should be adapted to context, and the setting of care modulates educational strategies. Where staff numbers are not an issue, direct patient and caregiver education should be the first option, with one-to-one dietary counseling delivered by dieticians, nurses and physicians during dedicated consultations, or within group educational programs (Table 3). Counseling should start from the assessment: understanding the person’s food habits and preferences and inquiring about food availability (grocery stores, fruit and vegetable markets near their homes), and the person’s food budget. Assessment is the key to success as it allows tailoring education to the single individual. Differences and dangers of UPF compared to fresh food should be explained, dedicating particular attention to instructing people on how to read food labels [137]. In fact, there are pitfalls even when buying what appear to be natural or plant-based food products: in the choice of vegetable drinks like coconut milk, rice or oat milk, it is important to read labels carefully as additives differ considerably from one brand to another.

Once the nutritional program has been established, strategies should be agreed with the patient, providing sample recipes and involving partners, family, and caregivers. The goal should be to propose options, not impose diets. There are plenty of different regimens that can be adopted to meet people’s preferences. Stepwise, gradual changes are more likely to be accepted and progressively integrated in daily life [15]. Ways should be found to vary food choice, avoiding monotony, and to introduce one day (or 1–2 meals) per week without restrictions on what the person eats (cheat day) [109,139]. If the context allows, grocery store tours and food baskets could be good ideas to demonstrate the feasibility of a healthy diet. Finally, changes with shared decisions should be gradually implemented.

Conversely, when time is the main issue, leaflets and infographics will help improve patient’s knowledge and awareness. Some examples have been published recently, and it is important to share educational materials with peers [137,140]. Table 4 presents some ideas about how to provide an educational program for people living with CKD.

## 11. Control Strategy

The exact frequency of clinical controls (nephrologic, dietetic) is not established, and even in the guidelines, this depends on the setting of care and the availability of equipment for anthropometric and other body composition measures. Ideally, it should be assessed during the first visit and repeated in longitudinal assessment to detect the first signs of PEW, which can occur even in overweight or obese individuals [2]. The panel was aware that while, in general, nephrologists are free to determine the frequency of their consultations, making an appointment with a dietician often requires a nephrology referral and, where visits are not reimbursed, the frequency of consultations also depends on economic constraints. Overall, there is general agreement that the frequency of consultations should increase in advanced CKD stages and with the progression of CKD.

The set of biochemical tests to perform in people following a protein-restricted diet is not agreed and likewise depends on the setting of care.

The panel highlighted a set of tests of higher relevance, besides kidney function tests, including nutritional and inflammatory markers (albumin, prealbumin, C-reactive protein, ferritin), anemia and iron status (blood cell count, iron, transferrin and transferrin saturation), and the assessment of vitamins levels (vitamin D, B9 and B12, plus vitamin A, E and C in selected cases), electrolytes and microelements (sodium, potassium, calcium, phosphorus, magnesium, uric acid, plus zinc and copper in selected cases).

According to the setting, the dosage may lead to the prescription of fortified grains, complex nutritional supplements or vitamins.

The panel highlighted a set of tests of higher relevance, besides kidney function tests, including nutritional and inflammatory markers (albumin, prealbumin, C-reactive protein, ferritin), anemia and iron status (blood cell count, iron, transferrin and transferrin saturation), and the assessment of vitamins levels (D, B9 and B12, plus A, E and C in selected cases), electrolytes and microelements (sodium, potassium, calcium, phosphorus, magnesium, uric acid, plus zinc and copper in selected cases).

Creatinine based formulas should be integrated, in particular in people on protein-restricted diets, with cystatin C measurements, if available; urea levels are likewise of interest, in particular in on-diet people Finally, 24 h urine collections should be performed, whenever possible, to estimate protein and sodium intakes.

## 12. The Dark Side of Dietary Management of CKD: What We Do Not Know

Specific patient populations deserve further studies. Limited evidence, from observational studies and RCTs, regards the emerging phenotypes of people living with CKD, i.e., people with obesity, diabetes, and old age, and suggestions mainly rely on expert opinions. Moreover, due to concerns about protein restriction, people living with diabetes have been intentionally excluded from the largest RCTs and available information mainly derives from observational studies [142,143,144]. The high prevalence of obesity poses further challenges, and its correction may be the priority, especially in people who are otherwise candidates for kidney transplantation.

In this regard, the expert panel agreed that LPDs and even VLPDs should be considered for highly motivated people living with kidney failure that refuse dialysis, regardless of age, as a tool to mitigate uremic symptoms [145,146]. This manuscript has an intrinsic limitation, being the result of the panel discussion obviously reflects the author’s expert opinions. Similarly, the issues presented in this review are the themes discussed during the meeting with no ambition to cover all the aspects of kidney nutritional management, such as differences in type and intake of processed and ultraprocessed food among different countries or continents. Nevertheless, our manuscript has the merit of presenting some practical considerations in the hope to clarify controversial aspects of CKD nutritional management and help nephrologists and dietitians implement the best level of care for their patients.

## 13. Final Considerations and Need for Research

Dietary management of CKD is not easy (Table 5). Dietary prescriptions need to be adapted to each individual and one single LPD that would suit everybody does not exist, as every person living with CKD is unique and has their own needs and preferences. We should also keep in mind that we do not eat nutrients, but we eat food, which is a mixture of nutrients, and the CKD plate needs to be considered as a whole, striking a balance between avoiding deficiencies, reaching the intended outcome of delaying CKD progression and dialysis need and respecting the patient’s food habits and their socioeconomic and cultural background. The experts involved in this discussion believe that plant-based diets should be promoted for the benefit of our people living with CKD and our planet [127]. Nevertheless, the dietary management of CKD requires multidisciplinary collaboration between nephrologists and dieticians. Further research is warranted on all aspects of dietary management of CKD, especially in light of the new drugs available to delay the progression of the disease to test their synergy with the diet [61]. Initiatives to support the dissemination and culture of healthy eating should be promoted at the general population level and in all nephrology settings.

Finally, more studies are needed on several issues: First of all, to verify the long-term effect and the impact of plant-based diets on CKD progression. Secondly, we need to better integrate the new concept of precision nutritional intervention, assessing the changes in the intestinal flora (such as short-chain fatty acid metabolism) to optimize dietary fiber intake strategies, and explore the role of fortified foods (ex. with higher vitamin D content) or specific plant compounds (such as flavonoids) in CKD management.

## Figures and Tables

**Table 2:** An example of how different formulas for estimating ideal body weight result in different dietary prescriptions.

	Actual Weight (kg)	Ideal Body Weight (Lorentz [79]) (kg)	Ideal Body Weight (Hamwi [77]) (kg)
	Woman: 62 years old; actual weight, 62 kg; height, 163 cm; BMI, 23.3 kg/m^2^		
	62	57	55
Energy (30 kcal/kg/day)	1860	1710	1650
Protein (0.6 g/kg/day)	37.2	34.2	33.0
	Man: 91 years old, actual weight, 80 kg; height, 175 cm; BMI: 26.1 kg/m^2^		
	80	69	72
Energy (30 kcal/kg/day)	2400	2070	2160
Protein (0.6 g/kg/day)	48.0	41.4	43.2

**Table 3:** Modality of educational interventions and topics. Adapted from [138].

Modality	Options	Topics Do Discuss
Setting	One-to-one Group Patient and family	Kidney physiology/pathology, treatment of CKD Medication management/adherence Nutrition and kidney diseases Pharmacological and medical protocols Nutritional counseling/dietician’s advice Lifestyle modification (e.g., weight loss, smoking) Exercise/program/information/participation Fresh food vs. ultra-processed food Self-management skills
Delivery technique	Face-to-face: slide, lectures, counseling, interviews Remote: e-learning, messages, social media, phone calls Written material: leaflets, books Other: medication charts, review of patient’s dietary journal	
Teaching method	Didactic Goal setting, dictated Goal setting, negotiated: self-management Situational problem solving: practical skills Other: support group discussions Workshops	

**Table 4:** Some ideas for nutritional education for people living with CKD.

Supermarket Guide	How to Buy, How to Choose Food [137]
Videos	Explaining food choices according to degree of food processing [137].
Personal adaptions	Food recipes adapted to the cultural background, identifying kidney-friendly options [109].
Cooking lessons	Workshops with professional cooks [141].
Group discussions	Identifying barriers and facilitators; involve expert patients.
Involve patient’s organizations	Establishing priorities and expectations.
Be the example	Discussing how you follow the recommendations patients are given.

**Table 5:** Dos and Don’ts in nutritional management of CKD, summary.

Dos ✓	Don’ts ✗
**Animal vs. vegetal proteins**	
Prescribe a plant-based diet in all CKD stages; regularly check nutritional status; periodically check for intake of all essential amino acids in the same meal (ideally) or at least in the same day, in particular in vegan diets. Consider occasionally including unrestricted meals. Consider supplementation with essential ketoacids and amino acids in vegan diets, both for simplifying management (0.6 g/kg/day diets) and making it possible to use VLPDs.	Do not limit prescription for fear of PEW. Do not prescribe a plant-based (vegan) or a VLPD without a detailed monitoring plan.
**Potassium**	
Prescribe plant-based diets in all CKD stages with regular checks of potassium and bicarbonate levels. Add potassium binders, provided that food sources are controlled (readily absorbed potassium salts are ubiquitous additives in ultra-processed food; they are not necessarily disclosed on food labels).	Do not limit prescription of plant-based diets in the case of high potassium levels, or fear of causing them. Do not prescribe a plant-based (vegan) or a VLPD without preparing a strict monitoring plan.
**Phosphorus**	
Keep in mind that a plant-based diet is a source of phosphorus whose bioavailability is lower than animal-based diets. Beware of ultra-processed foods, as readily absorbed phosphate salts are ubiquitous additives in ultra-processed food and they are not necessarily disclosed on food labels.	Do not limit prescription of plant-based diets in the case of high phosphate levels, or fear of causing them.
**Ideal body weight**	
Consider each person living with CKD’s body composition, weight trajectory and perspectives when approaching the issue of real or ideal body weight. Consider real body weight at least below a BMI of 30 kg/m^2^. Evaluate obese people living with CKD individually and review prescriptions in case of modifications of body weight.	Do not use a formula to calculate ideal or adjusted body weight without a previous critical appraisal of the individual person living with CKD.
**Energy intake and the risk of protein energy wasting**	
When starting nutritional management of people living with CKD, always start from energy intake, modulating it according to the person’s nutritional status. A plant-based diet is compatible with high energy intake, and if necessary, can be combined with energy-rich low-protein supplements.	In people living with CKD with evidence of PEW, do not restrict protein intake, mainly from vegetable sources, at least until energy needs are met. Avoid a high-protein intake to prevent protein hypercatabolism, potentially leading to acidosis, hyperkalemia, hyperphosphatemia and intestinal dysbiosis.
**Supplemented very low protein diets**	
Prescribe VLPDs to both young and elderly people living with CKD. They are in essence plant-based. Especially in people living with CKD on unrestricted baseline diets, proceed stepwise, both with quality changes (from omnivorous to vegan) and protein intake.	Do not prescribe a severe protein restriction without supplementation and without close monitoring. Do not prescribe sVLPD in malnourished people living with CKD, especially if the etiology of PEW is uncertain. Do not use the same doses when supplementation is an add-on treatment in moderately protein-restricted diets. Consider a lower pill burden, instead.
**Ultra-processed foods**	
Pay attention to the quality of food and minimize the use of ultra-processed foods.	Do not reduce plant-based food, for example, in the case of hyperkalemia, until you have excluded regular use of UPFs and educated people living with CKD to limit their use.

## Data Availability

No new data was produced for this review.
